# Agile parallel bioinformatics workflow management using Pwrake

**DOI:** 10.1186/1756-0500-4-331

**Published:** 2011-09-08

**Authors:** Hiroyuki Mishima, Kensaku Sasaki, Masahiro Tanaka, Osamu Tatebe, Koh-ichiro Yoshiura

**Affiliations:** 1 Department of Human Genetics, Nagasaki University Graduate School of Biomedical Sciences, 1-12-4 Sakamoto, Nagasaki, Nagasaki, Japan; 2 Nagasaki University Global Center of Excellence Program, 1-12-4 Sakamoto, Nagasaki, Nagasaki, Japan; 3 Center for Computational Sciences, University of Tsukuba, 1-1-1 Tennodai, Tsukuba, Ibaraki, Japan; 4 Core Research for Evolutional Science and Technology, Japan Science and Technology Agency, 4-1-8 Honcho, Kawaguchi, Saitama, Japan; 5 Departmentent of Computer Science, Graduate School of Systems and Information Engineering, University of Tsukuba, 1-1-1 Tennodai, Tsukuba, Ibaraki, Japan

## Abstract

**Background:**

In bioinformatics projects, scientific workflow systems are widely used to manage computational procedures. Full-featured workflow systems have been proposed to fulfil the demand for workflow management. However, such systems tend to be over-weighted for actual bioinformatics practices. We realize that quick deployment of cutting-edge software implementing advanced algorithms and data formats, and continuous adaptation to changes in computational resources and the environment are often prioritized in scientific workflow management. These features have a greater affinity with the agile software development method through iterative development phases after trial and error.

Here, we show the application of a scientific workflow system Pwrake to bioinformatics workflows. Pwrake is a parallel workflow extension of Ruby's standard build tool Rake, the flexibility of which has been demonstrated in the astronomy domain. Therefore, we hypothesize that Pwrake also has advantages in actual bioinformatics workflows.

**Findings:**

We implemented the Pwrake workflows to process next generation sequencing data using the Genomic Analysis Toolkit (GATK) and Dindel. GATK and Dindel workflows are typical examples of sequential and parallel workflows, respectively. We found that in practice, actual scientific workflow development iterates over two phases, the workflow definition phase and the parameter adjustment phase. We introduced separate workflow definitions to help focus on each of the two developmental phases, as well as helper methods to simplify the descriptions. This approach increased iterative development efficiency. Moreover, we implemented combined workflows to demonstrate modularity of the GATK and Dindel workflows.

**Conclusions:**

Pwrake enables agile management of scientific workflows in the bioinformatics domain. The internal domain specific language design built on Ruby gives the flexibility of rakefiles for writing scientific workflows. Furthermore, readability and maintainability of rakefiles may facilitate sharing workflows among the scientific community. Workflows for GATK and Dindel are available at http://github.com/misshie/Workflows.

## Background

The concept of workflows has traditionally been used in the areas of process modelling and coordination in industries [1]. Now the concept is being applied to the computational process including the scientific domain. Zhao *et al*. found that general scientific workflow systems are employed in and applied to four aspects of scientific computations: 1) describing complex scientific procedures, 2) automating data derivation processes, 3) high-performance computing (HPC) to improve throughput and performance, and 4) provenance management and query [2]. Although naïve methods such as shell scripts or batch files can be used to describe scientific workflows, the necessity of workflow systems arises to satisfy the four aspects mentioned above. Therefore, full-featured scientific workflow systems including Biopipe [3], Pegasus [4], Ptolemy II [5], Taverna [6], Pegasys [7], Kepler [8], Triana [9], Biowep [10], Swift [11], BioWMS [12], Cyrille2 [13], KNIME [14], Ergatis [15], and Galaxy [16] have been applied in the bioinformatics domain. Their features, however, have some disadvantages for actual practices in bioinformatics. It is not always easy to describe actual complex workflows using graphical workflow composition, and some workflow language formats, such as XML, are not very readable for humans. Moreover, these workflow systems often require wrapper tools, which are called "shims", to handle third-party unsupported existing code or data sources [17,18]. This sometimes obstructs quick deployment of newer tools. In actual bioinformatics projects, we realized that scientific workflow systems often require quick deployment of cutting-edge software to implement new algorithms and data formats, frequent workflow optimization after trial and error and in following changes in computational resources and the environment. The agile software development method considers similar problems in software development projects. Kane *et al*. summarized this by stating that "Agile is an iterative approach to software development on strong collaboration and automation to keep pace with dynamic environment", and "Agile methods are well suited to the exploratory and iterative nature of scientific inquiry" [19]. Therefore, scientific workflow systems require both rigidity in workflow management and agility in workflow development.

One of the traditional solutions for balancing the two aspects of a workflow system is the make command, a standard build tool in the Unix system. The make command interprets a Makefile, which defines dependencies between files in a declarative programming manner, and then generates the final target by resolving dependencies, by only executing out-of-date steps. This approach has been extended to cluster environments such as GXP make [20]. However, the make-based approach has limitations in describing scientific workflows because it is intended for building software. For example, it is difficult to describe the "multiple instances with *a priori *runtime knowledge" pattern, which is one of the workflow patterns defined by Van der Aalst *et al*. [1], in makefiles without external tools. In this pattern, the number of instances is unknown before the workflow is started, but becomes known at some stage during runtime. In other words, this situation requires dynamic workflow definition at runtime. This pattern appears frequently in scientific workflows as well as embarrassingly parallel problems. Introduction of internal domain specific languages (DSLs) to workflow description is an approach to overcome this limitation. Internal DSLs are implemented as libraries of the host languages. Thus, an internal DSL retains the descriptiveness of the host language.

Introduction of the internal DSL into make-like workflow systems has been shown in object-oriented scripting languages including Python [21] and Ruby [22]. An implementation in Python is Ruffus [23], which is a scientific workflow system supporting execution limited to out-of-date stages, dynamic workflow definition, flowchart generation, and parallelism. PaPy [24], another workflow system in Python, was implemented with a modular design and offers parallel and distributed workflow management. On the other hand, the Ruby programming language also has a greater affinity to the internal DSL approach because of its flexible syntax, including omissible parentheses and a code-block grammar [25]. Rake [26] is a 'Ruby Make', which is a build tool with workflow definition implemented as an internal DSL in Ruby and a standard library of Ruby version 1.9 or later. Rake supports execution of workflows limited to out-of-date stages and dynamic workflow definition during workflow execution. The following is a simple example of a workflow definition file, a Rakefile:

1: CC = "gcc"

2: rule '.o' = > '.c' do |t|

3:   sh "#{CC} -c #{t.source}"

4: end

5: file "sample" = > ["sample.o"] do |t|

6:   sh "#{CC} -o #{t.name} #{t.prerequisites}"

7: end

8: task :default = > "sample"

This example defines a workflow to generate an executable sample from sample.c via sample.o. If sample.c is out-of-date, i.e., older than sample.o, Rake skips compiling sample.c and just links sample.o to generate sample. Note that the grammar of the rakefile is fully compatible with that of Ruby.

Recently Tanaka and Tatebe developed Pwrake [27], a parallel workflow extension of Rake. Pwrake has been demonstrated to be a flexible scientific workflow system in the astronomy domain [28]. It interprets rakefiles that are fully compatible with Rake. Pwrake supports parallelism by automatically detecting parallelizable tasks and executing them via SSH connections. Pwrake generates a flowchart as a directed acyclic graph in the DOT language, which is then visualized by software such as Graphviz [29]. Although we focus on workflow management using a local multiprocessor and multicore environment, Pwrake can be used with computer clusters together with the support of a distributed filesystem such as NFS. Pwrake is especially designed for scalable parallel I/O performance using the Gfarm global distributed filesystem [28,30].

In this paper, we show agile workflow management using Pwrake in the bioinformatics domain.

## Implementation

### Rakefiles

**Figure 1:**
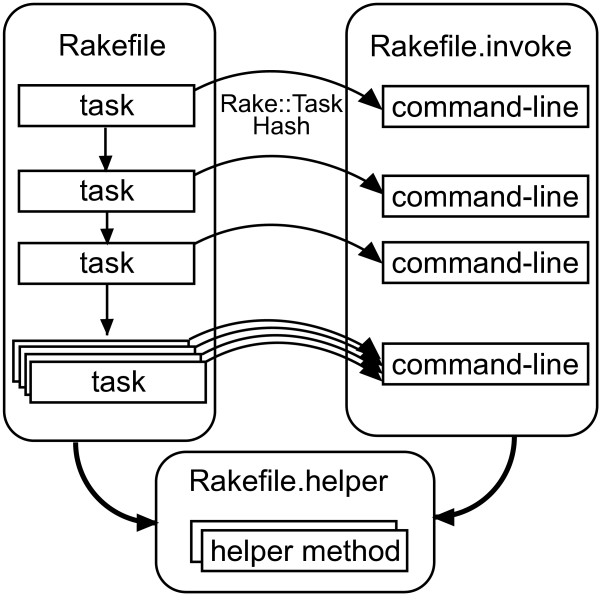
**Structure of distinct rakefiles**. A Rakefile file consists of task dependency descriptions. Tasks may be executed in parallel, if possible automatically. The rakefile.invoke file defines a class of the RakefileInvoke module. This class defines class methods to invoke command-lines and constants of command paths and parameters. Tasks in the rakefile call methods with an instance of the Rake::Task class and a hash containing additional parameters for invoking the command-line. The Rakefile.helper file defines helper methods to simplify descriptions in the Rakefile and Rakefile.invoke files.

In actual bioinformatics workflow development, we found that the scientific workflow development iterates over two phases, the workflow definition phase and the parameter adjustment phase. The former focuses on the functional combination and order of tasks, while the latter focuses on the optimization of command-line parameters for invoking tools. We therefore, designed separate rakefiles corresponding to these two phases. Task dependencies are defined in Rakefile, while command-line programs and parameters are defined in Rakefile.invoke. To simplify the description, we also implemented a file to define helper methods, Rakefile.helper (Figure 1).

Rakefile is the main and default task definition file. It loads two other rakefiles, sets target filenames in constants, and declares task dependencies. Other rakefiles are loaded by the Kernel#load method to enable reloading to reflect changes immediately.

Rakefile.invoke defines a class with a unique name in the RakefileInvoke module. In the class, paths to commands and common files, as well as adjustable parameters are set to constants. It also defines methods to invoke command-lines using FileUtils#sh methods. These methods are defined as singleton methods (eigenmethods) of the class. This is an internal DSL technique in Ruby to enable invocation in rakefiles as in "RakefileInvoke::Gatk::command t, opts", where t is an instance of the Rake::Task class and opts is a hash object containing the optional information to invoke commands. Rakefile.helper defines helper methods to simplify the rakefile descriptions. For example, the suffix method in the top level allows the replacement of the filename suffix using expressions with arrows. Additionally, Pwrake requires a nodefile to specify hostnames and maximum numbers of processes to be submitted via SSH connections. A nodefile declaring a local machine that can execute 16 processes simultaneously is set as "localhost 16".

Command-lines to start the workflow using Rake and Pwrake are "rake" and "pwrake NODEFILE = nodefile", respectively. By default, Rake and Pwrake load the file called "Rakefile" in the current directory. Rakefiles are usually placed in the topmost directory in a project file tree. To simplify provenance management, we recommend that each project file tree has its own copy of the rakefile.

### Example workflows

To demonstrate the workflows described in Pwrake rakefiles, we implemented two kinds of workflows for the Genome Analysis Toolkit (GATK) [31,32] and Dindel [33] using rakefiles. Both GATK and Dindel have been used in whole genome sequencing projects including the 1000 genomes project [34]. We selected GATK and Dindel as typical examples for sequential and parallel workflows, respectively. Furthermore, we implemented a combined workflow loading externally defined GATK and Dindel workflows to show the modularity thereof.

### The GATK workflow

**Figure 2:**
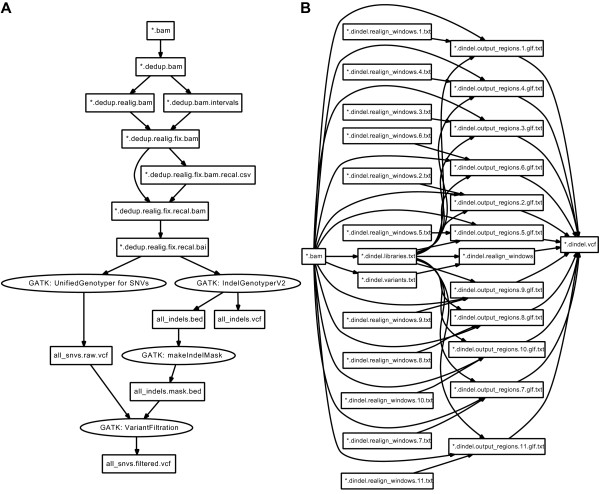
**Directed acyclic graphs of GATK and Dindel workflows**. Directed acyclic graphs to describe GATK (A) and Dindel (B) workflows are generated by Pwrake, manually adjusted, and visualized using Graphviz. (A) depicts a workflow from a *.bam file to an all_snvs.filtered.vcf, while (B) depicts a workflow from a *.bam file to a *.dindel.vcf file. Tasks to process the *.dindel.realign_windows.N.txt files are dynamically generated during the embarrassingly parallel stage (stage 3) depending on *.dindel.libraries.txt and *.libralies.txt. Prior to stage 3, an option "--numWindowsPerFile 1000" is applied to makeWindows.py to generate 11 files containing windows. File *.didndel_reagion_window is a dummy file representing all the *.dindel.realign_windows.N.txt files.

GATK is a program suite written mainly in Java to process mapped reads obtained from massively parallel sequencing data to detect genetic variants including single nucleotide variants (SNVs). The GATK development team offers several recommended workflows depending on the samples and analyses. We implemented their 'better' workflow (Figure 2A). In Rakefile, the Rakefile::Gatk class defines constants indicating the target files in each step of the workflow. These constants are used to define the :default task to obtain the final product of the workflow. In Rakefile.invoke, the RakefileInvoke::Gatk class defines constants indicating the file paths to executables and downloaded public data files, such as the reference genome sequence and dbSNP data. These help the workflow configuration in other environments and improve readability. The class also defines methods to execute command-lines for each step in the workflow.

### The Dindel workflow

Dindel is a suite of tools for detecting small genetic insertions and deletions (indel) from massively parallel sequencing data. The overview of the rakefile structure for GATK and Dindel is the same; however, a Dindel workflow is a good example of a parallel workflow using the dynamic task definition (Figure 2B). Such a workflow generates many intermediate files. In the authors' experience, one human exome generates more than 300 "window" files, where each window file can contain a maximum of 1000 windows. These intermediate window files are named systematically; however, the number of window files is unknown prior to the workflow execution. A rakefile can describe this situation using a dynamic task definition. Furthermore, Pwrake can automatically detect tasks that can be executed in parallel. The following is an example of dynamic task definition codes based on the stage 3 definition of the Dindel workflow in Rakefile and Rakefile.invoke.

1: # Rakefile

2: task :stage3 = > :stage2 do

3:   Rakefile::Dindel::BAM.each do |bam|

4:      prefix =

5:         bam.sub(/\.bam$/, ".dindel.realign_windows")

6:      FileList["#{prefix}.*.txt"].each do |f|

7:         target = f.sub(/\.realign_windows\./,

9:                    ".output_regions.").

6:                sub(/\.txt$/, ".glf.txt")

7:         prerequisites =

8:           [f,

9:            f.sub(/\.dindel\.realign_windows\..*/, ".bam"),

10:           f.sub(/\.dindel\.realign_windows\..*/,

11:                 ".dindel.libraries.txt"),]

12:         file target = > prerequisites do |t|

13:           RakefileInvoke::Dindel.dindel_stage3 t

14:         end

15:         file :stage3_invoke = > target

16:        end

17:      end

18:       (task :stage3_invoke).invoke

18: end

1: # Rakefile.invoke

2: def dindel_stage3(t)

3:   sh [DINDEL,

4:      "--analysis indels",

5:      "--doDiploid",

6:      "--bamFile #{t.prerequisites[1]}",

7:      "--ref #{REFERENCE}",

8:      "--varFile #{t.prerequisites[0]}",

9:      "--libFile #{t.prerequisites[2]}",

10:      "--outputFile #{t.name.sub(/\.glf\.txt$/, "")}",

11:      "1 > #{t.name.sub(/\.glf\.txt$/, "")}.log 2 > &1",

12:      ].join(" ")

13: end

In this sample rakefile, the :stage3 task expects that the previous task :stage2 generates files that are named *.dindel.realign_windows.N.txt, where N is the serial number of the intermediate file. The maximum value of N is unknown prior to execution of the :stage2 task. The dependency of the following stages can be defined using the task name :stage3.

Pwrake automatically detects that :stage3 consists of independent file tasks and executes them as an embarrassingly parallel stage. In the :stage2 definition in Rakefile.invoke, the granularity of parallelism can be defined by the "--numWindowsPerFile" option of makeWindows.py. For the exome dataset aligned to chromosome 21, we used 1000 and 1 for this option and obtained 11 and 3381 intermediate realign_windows files, respectively.

### Combination of rakefiles

Existing rakefiles can be combined by being loaded into another rakefile. Constants and methods defined in rakefile.invoke files have independent namespaces. Moreover, a task with the same identifier, such as the :default task, can be defined multiple times and thus can be appended. Pwrake and Rake do not overwrite, but append the files. For example, a rakefile to define GATK and Dindel workflows simultaneously simply contains the following:

1: load "../GATK/Rakefile"

2: load "../Dindel/Rakefile"

## Results

### Performance

To evaluate the performance of the GATK and Dindel workflows, we analysed publicly available short read sequence data using a Linux system that can execute 16 concurrent threads (2 processors × 4 cores with hyper-threading). Whole genome sequencing data [35] obtained from a HapMap [36] JPT sample NA18943 was used as the test dataset. The dataset was mapped to the GRCh37 referential genome sequence using the Burrows-Wheeler Alignment tool (BWA) [37] to generate a SAM file [38]. The SAM file was converted to a BAM file using Picard [39]. Reads mapped on chromosome 21 were used as initial data for both the GATK and Dindel workflows. We executed both Rake and Pwrake with the same rakefiles to compare the performance with parallelism. The wall-clock times for the GATK workflows executed by Rake and Pwrake were almost identical (approximately 12.0 min). We assume that this is due to the high sequentiality of the workflow. For the Dindel workflow, we assessed different parallelism granularities. When the task was divided into 11 processes in stage 3, the Dindel workflow executed by Pwrake was 2.6 times faster (approximately 6.0 min) than that by Rake (approximately 15.5 min). When the task was divided into 3381 processes in stage 3, the Pwrake execution was 4.6 times faster (approximately 4.0 min) than the Rake execution (approximately 18.3 min). While the ideal parallel acceleration efficiency was 16 times for our computer environment, the actual efficiency differed. These results can be explained by the fact that the required CPU-time to finish each process was uneven, and a few heavy processes were bottlenecks in the workflow execution. This is a limitation of process-based parallelism because of the relatively coarse parallelization granularity.

### Agility in workflow development

A characteristic of agile software development is the iterative development process. We introduced an agile scientific workflow development that employed the iteration of two developmental phases, i.e., the workflow definition phase and the parameter adjustment phase. In each phase, our implementation of distinct rakefiles enabled the separate files to be modified. This separation increased efficiency in the iterative development.

Here, we show an example of the iterative development in our GATK workflow. In the workflow definition phase, we focus on describing a task dependency in a rakefile as shown below:

1: rule '.dedup.bam.intervals' = >

2:   [ suffix_proc(".bam.intervals" = > ".bam") ] do |t|

3:   RakefileInvoke::Gatk.gatk_realigner_target_creater t

4: end

Next, in the parameter adjustment phase, we focus on describing command-line parameters for invoking external tools in the rakefile.invoke such as the following:

1: def gatk_realigner_target_creater(t)

2:  sh [Java,

3:     "-Xmx#{JavaMemory}",

4:     "-Djava.io.tmpdir = #{JavaTempFile}",

5:     "-jar #{GATK_JAR}",

6:     "-T RealignerTargetCreator",

7:     "-R #{REFERENCE}",

8:     "-o #{t.name}",

9:     "-I #{t.source}",

10:     "-D #{DBSNP}",

11:     RakefileInvoke::Gatk::INTERVAL_OPTION,

12:     " > #{t.name}.log 2 > &1",

13:     ].join(" ")

14: end

Note that all constants with names starting with uppercase letters are defined at the top of the file, rakefile.invoke. The next iteration starts with the workflow definition phase again to extend the workflow. Modification or optimization after the workflow has completed can be achieved by iterating the same two phases using two distinct files. Separating the rakefiles simplifies finding files and places to be modified.

### Procedure to describe new workflows

As a summary of the agile workflow development, the general procedure for describing new workflows in Pwrake is given below.

1) Workflow definition phase. Describe file dependencies in Rakefile.

1: task "output.dat" = > "input.dat" do |t|

2:   RakefileInvoke::generate_target t

3: end

2) Parameter adjustment phase: Define the RakefileInvoke::generate_target method in Rake.invoke.

1: module RakefileInvoke

2:   def generate_target(t)

3:      sh "command-line #{t.prerequisite} > #{t.name}"

4:   end

5: end

3) Iteration of phases. Parameter adjustments require modifications to Rakefile.invoke only. Similarly, changes in file dependencies require modification to Rakefile only.

## Discussion

### Advantages in workflow execution

Workflows involving actively developed software packages, such as GATK, require frequent updates of details, such as combinations of data and programs, recommended parameters, and command-line options. Thus, well-organized workflow management helps GATK users to follow updates and process their data in improved workflows. A GATK workflow consists of multiple steps and takes a relatively longer time to finish. Pwrake has advantages of continuous execution of workflow tasks and selective task execution to ignore already executed tasks. Such ignorable tasks can be obtained from unexpected workflow suspension. Thus far, Pwrake cannot automatically remove output files containing partial results; such files have to be removed manually prior to restarting the workflow.

For the Dindel workflows, the parallelism offered by Pwrake improved performance. The parallelization model of Pwrake is process-based. Parallel programs based on technologies such as message passing interface (MPI) [40] enable efficient parallelization with fine granularity. However, scientists implementing bioinformatics software often focus not on parallelization, but on the novel implementation methodology. Therefore, process-based parallelization using non-parallel programs is a realistic solution and still has the advantage [41]. Furthermore, process-based parallelization can be efficient enough for embarrassingly parallel problems that can easily be separated into independent tasks and executed in parallel. For example, a stage in the Dindel workflow creates multiple intermediate files. Processes using these files as input are independent and do not need to communicate with each other. This stage is a typical embarrassingly parallel problem. Although the GATK framework supports the functional programming concept of MapReduce [42] and parallelism in the GATK framework is expected to improve its performance, it has only been supported to a limited extent by GATK components to date. Therefore, Pwrake still has the advantage with respect to parallelism.

### Workflow description flexibility

One of the advantages of using an internal DSL is that the power of the host language is also available in the DSL scripts. The rakefile description is an internal DSL in Ruby, which is a programming language with a shallow learning curve for biologists [43]. Thus, rakefiles can make full use of the control flow features of Ruby, as well as the rich libraries for text processing, file manipulation, network access, and so on. In particular, the BioRuby [44] library offers highly abstracted data processing methods for bioinformatics.

### Sharing workflows

One of the key characteristics of agile software development is strong collaboration among all the people involved in the project. This can be accomplished naturally in projects in small laboratories. However, the nature of science is a global collaboration. Indeed, efforts to share and reuse workflows in the science community, such as the myExperiment project [45] and Wf4Ever [46], have already been started. From this point of view, the simplicity and readability of the rakefile DSL are advantageous, and improvement of helper methods to standardize the scripting style on the "Do not Repeat Yourself (DRY)" principle may enhance the advantages.

## Conclusions

We have shown an appreciation of Pwrake as an agile parallel workflow system suitable for the bioinformatics domain using examples of GATK and Dindel workflows. Pwrake is able to invoke command-line tools without any "shims", define tasks dynamically during the workflow execution, and invoke tasks automatically in parallel. Separating a rakefile into two files for the workflow definition phase and the parameter adjustment phase increases the efficiency of the iterative workflow development. The nature of scientific projects is explorative and iterative. This is also a characteristic of agile software development. Another aspect of agile development, the reliance on the strong collaboration, may be enhanced by sharing and reusing workflows among the scientific community by taking advantage of the simplicity, readability and maintainability of rakefiles.

## Availability and requirements

Project name: Workflows

Project home page: http://github.com/misshie/Workflows

Operating system(s): Platform independent

Programming language: Ruby 1.9.1 or higher

Other requirement: Pwrake or Rake

License: the MIT license

Any restrictions for use by non-academics: none

### Availability of supporting data

Sample short read data for workflow evaluation: http://trace.ddbj.nig.ac.jp/DRASearch/experiment?acc=DRX000358

## List of abbreviations used

HPC: high-performance computing; DSL: domain specific language; GATK: Genome Analysis Toolkit; SNV: single nucleotide variant; BWA: Burrows-Wheeler Alignment tool; MPI: message passing interface; DRY: do not repeat yourself.

## Competing interests

The authors declare that they have no competing interests.

## Authors' contributions

HM conceived the study, implemented the workflows, and co-authored the manuscript. KS implemented the workflows. MT and OT developed Pwrake and evaluated the details of the workflows and the computational performance. KY conceived the study and co-authored the manuscript. All authors read and approved the final manuscript
